# Late life depression and concepts of aging: an emerging paradigm

**DOI:** 10.3389/fmed.2023.1218562

**Published:** 2023-08-09

**Authors:** Jeremy M. Jacobs, Lea Baider, Gil Goldzweig, Eli Sapir, Yakir Rottenberg

**Affiliations:** ^1^The Jerusalem Institute of Aging Research, Hadassah Medical Center and Faculty of Medicine, Hebrew University of Jerusalem, Jerusalem, Israel; ^2^Department of Geriatric Rehabilitation and the Center for Palliative Care, Hadassah Medical Center and Faculty of Medicine, Hebrew University of Jerusalem, Jerusalem, Israel; ^3^Oncology Institute, Assuta Medical Center, Tel Aviv, Israel; ^4^School of Behavioral Sciences, Academic College of Tel Aviv-Yaffo, Tel Aviv, Israel; ^5^Department of Radiation Oncology, Samson Assuta Ashdod University Hospital, Ashdod, Israel; ^6^Department of Oncology, Hadassah Medical Center and Faculty of Medicine, Hebrew University of Jerusalem, Jerusalem, Israel

**Keywords:** late life depression, aging models, brain aging, intrinsic capacity, resilience

## Abstract

Late life depression (LLD) is an emerging challenge, and recognized as a significant barrier to long-term healthy aging. Viewed within the context of the medical/biological model, advances in brain sciences over the last several decades have led to a deeper understanding of the biology of LLD. These advances in current knowledge include the description of aging brain pathophysiology; the biology and biochemistry of neurotransmitters; the correspondence between changes in neurological structure, function, and neural network; the description of neural, hormonal and inflammatory biomarkers; and identification of typical phenotypic subtypes of LLD. Despite these advances, current treatment of LLD, which remains largely pharmacological with accompanying cognitive and behavioral interventions, has poor success rate for long-term remission among older people. A wider perspective, in keeping with several emerging aging concepts, is suggested as an alternative framework within which to view LLD. A growing body of research supports the important role in LLD of frailty, resilience, intrinsic capacity, and functional integrity. Similarly, important social determinants need to be addressed in the etiology of LLD, rooted largely in negative stereotypes of aging, with consequent repercussions of reduced participation and inclusion, growing social isolation, with loss of identity, meaning and hope. This perspective suggests the importance of a wider integrative conceptualization of depression, set against a background of emerging aging concepts.

## Introduction

The rising prevalence and incidence of Late Life Depression (LLD) among people aged over 65 years in general, and among the oldest old in particular, is increasingly recognized as an emerging challenge of global magnitude (1, 2). Indeed, current World Health Organization (WHO) recommendations include the alleviation and improvement of mental health, particularly depression, as an important sustainable goal in the maintenance of long-term healthy aging (3). In order to achieve this goal, a clear conceptual understanding and definition of LLD is required, As will be discussed in this “Perspective,” an attempt to conceptualize LLD highlights numerous current shifts and developments in our understanding of aging itself. Indeed, an understanding of LLD exemplifies several current emerging concepts in aging, and the shifting paradigms in which several common geriatric issues are being re-evaluated (4).

## Late life depression: the historical medical model

### Brain pathology

Firmly rooted in a biologically driven disease model, the fruits of research over the last several decades have led to a large body of evidence-based advances in our understanding of LLD. Detailed description of numerous pathogenomic changes in pathophysiology; the biology and biochemistry of neurotransmitters and their pathways; structural and functional brain changes alongside neural networks; neural, hormonal and inflammatory biomarkers; as well as clusters of behavioral, cognitive and functional phenotypic subtypes-all these lend themselves to an increasingly complex yet detailed biological nomenclature of LLD. A recent expert review concerning the wide range of biological factors and aging processes influencing LLD, presents a strong case to support the bi-directional view, whereby LLD in and of itself further accelerates the aging process (5). Similarly, the aging individual’s degree of resilience or vulnerability play an important modulating role in the biology of depression. Accordingly, evidence suggests that optimal treatment for depression is to be achieved through augmentation of somatic therapies with brief focused psychotherapy and cognitive training, alongside interventions to improve social connectedness, movement, and sensory function (5).

### Phenotypes

Several biological phenotypic subtypes of LLD have been consistently described, each hypothesized to reflect distinct aspects of biological aging. Thus, for example three broad areas have been identified, namely cerebrovascular aging; inflammation and senescence with dopamine depletion; and oxidative stress with mitochondrial dysfunction and energy dysregulation. These, in term, have been hypothesized to bear a clinical correspondence with three phenotypic subtypes of LLD: the “depressed patient with executive dysfunction”; the “inflamed-slowed” depressed patient; and the “frail-fatigued” depressed patient, respectively (2). Coexisting neurodegenerative processes further contribute to these suggested phenotypes, with additional abnormalities in cognitive impairment, reduced processing speed, impaired speech fluency, abnormal gait characteristics, as well as patterns suggestive of the evolving categories of cognitive fatigue and reduced cognitive reserve (6, 7).

### Neural networks

Advances in the delineation of neural networks have also contributed to an understanding of depression and associated changes observed both within and between key neural networks. Aided by technological innovations including functional MRI, neuro imaging, and systems analysis, recent evidence supports the linkage between both structural and functional aging brain changes, with several abnormal neural networks consistently observed to play a prominent role in depression. Thus, for example, LLD has been shown to be associated with abnormalities in the ventral limbic affective system (dysphoria), frontal striatal reward network (anhedonia), abnormal default mode network connectivity (depressive rumination), and the dorsal cognitive control network (cognitive deficits with diminished top-down control of negative thoughts and emotions) (8, 9).

### Complex networks

Originating in the study of frailty and multi-morbidity, the concept of aging has been hypothesized to reflect the sum consequences of declining complex systems and networks within the overall biology of aging of the entire human organism. Thus, research has linked not only physical frailty, but also LLD to the accumulated impairments, dysregulation and decline across a wide spectrum of biological networks. Such an approach would lend well to understanding the increased somatization typical of LLD, when viewed against the background of increasing age-associated multimorbidity and symptom complexity (10, 11).

The emerging understanding of the biology of LLD serves to drive a number of potential avenues for future research, aimed at the optimal control and prevention of neurovascular risk, reduced oxidative stress, and an accompanied decline in rate of brain aging. Improved clinical assessment used to identify phenotypic subtypes of LLD would be useful in order to help guide specific tailored pharmacological antidepressant medications, in conjunction with personalized behavioral interventions, brief psychotherapy, intervention to alleviate loneliness, cognitive training and exercise, and improved vision and hearing loss (5). Research into novel drugs, designed according to the pathogenomic biochemical and neurotransmitter changes would, it is proposed, serve to increase the fairly poor response rate to antidepressant medication for LLD, especially among the oldest old.

## An alternative view of LLD

As attractive and persuasive as the medically driven model may appear, nevertheless it remains a fact that numerous people with brain pathology remain depression free, while around two thirds of patients with LLD will remain unresponsive to drug treatment (2).

The etiology of LLD is clearly multi factorial, and in contrast to the disease models driving much of research, it seems necessary and appropriate to consider LLD within a far wider, integrative perspective, within the context of several emerging concepts of aging. Thus, for example intrinsic capacity, physical and cognitive reserve, resilience, alongside the maintenance of physical activity, in conjunction key social and psychological factors are all proving to be important drivers not only for successful aging, but also significant determinants of remaining free from depression despite advancing age.

### Resilience

Resilience has traditionally been described in psychosocial research as the capacity to maintain or regain well-being during or after adversity (12). Physical resilience has yet to gain a consensus definition, however common existing working definitions generally include the ability to resist or recover from functional decline following health stressors. Incorporated into models of successful healthy aging (13), resilient individuals maintain their ability to successfully contend with adversity, maintaining functional integrity, as well as a preserved sense of well-being. Attempts to operationalize resilience (14), specifically in the context of resilience to LLD, have generated a characteristic set of psychosocial and biological variables, which include a range of inherent attributes, processes, and outcomes. Thus, a positive set of attributes including temperament, level of attachments, personality; beliefs and coping strategies; as well as social and lifestyle factors have been implicated in remaining free from depression during advanced age (5, 15).

### Intrinsic capacity

Intrinsic Capacity, closely related yet distinct from resilience, is an emerging concept that the WHO recently proposed as one of the key driving force behind successful aging. Within the ongoing interface and interaction between individual and environment, it is one’s degree of intrinsic capacity that mediates and ultimately determines the degree of successful aging (16).

### The WHO international classification of function, disability, and health

It is useful to envisage Intrinsic capacity in relation to the wider conceptual framework of the WHO’s International Classification of Function, Disability, and Health (ICF) (17). Replacing the previous linear biological/medical/disease driven model of illness, the ICF conceptualizes the person’s level of function and activity as the outcome and overall summation of the simultaneous ongoing interaction between an individual’s Health conditions, Body functions, Participation, Environment, Personal Factors, and Participation. Recent aging theorists attempting to define intrinsic capacity have suggested that it closely correspond with and shares much in common with the “Body Functions” in the ICF model. Attempts to operationalize a measurable construct of intrinsic capacity have repeatedly suggested that it incorporates the broad domains of cognition, locomotion, sensory integrity (particularly hearing and vision), vitality, and psychological capacity (16).

Among the large body of psychological research into depression, ample evidence exist showing the robust association between LLD and numerous aspects of both the ICF model, as well as close correspondence with the more recent attempts to operationalize and measure intrinsic capacity.

### Frailty

Closely related to intrinsic capacity, yet distinct, is the concept of frailty (18). In some ways the mirror opposite of intrinsic capacity, frailty recognizes the qualitative heterogeneity of aging, and attempts to quantify the vulnerability of the individual, with the goal of understanding different trajectories of either health and successful aging, or more commonly, prognosis of decline and poor outcomes (19). Numerous approaches to frailty exist, which include not only physical but also cognitive and social frailty, and a growing body of literature exists linking frailty with correlates of well-being and depression in late life (20–23).

### Ageism

The pervasive nature of ageism is not to be underestimated, and the older person’s changing role in society is frequently perceived as negative, with growing objective measures of dis-inclusion, declining social recognition and socio-economic status, reduced levels of participation and engagement, and rising levels of social isolation and loneliness (24, 25). Consistent associations with LLD have been described across numerous social and psychological elements (26). Among these elements are loss of social identity and meaning, demoralization, reduced productivity and consequent loss of employment identity, life roles, social isolation within the family and society, physical and psychological dependence, limited availability and accessibility of community resources (transportation, companionship, home help) and presence or absence of support from family, friends and the close-knit community. Relevant risk factors include social isolation and loneliness, stress (including caregiver stress), sleep problems, lack of physical activity, functional limitations, as well as additional issues of addiction and alcoholism. Similar consistent findings support the strong associations and often causative relationship with LLD across a range of factors at the psychological and cognitive level: the individual’s perceived self-worth, self-perceived health (27–31) and degree of helplessness. In contrast, there is a wealth of literature concerning successful and depression free aging, associated with domains of faith and religious belief (32, 33), hope, meaning, purpose, existential meaning and life-satisfaction (34, 35). Taken collectively, the psychobiological factors of resilience form a critical set of attributes and assets which serve to buffer and protect the individual from LLD (15).

## Avenues for change

A deeper understanding of the biology of LLD, in keeping with the specific vascular, oxidative, and inflammatory etiology, suggest the possibility of improved diagnostic and clinical classification of LLD, according to the phenotypic sub-types of executive-dysfunction, cognitive-motoric impairments, frailty, fatigability, and slowed inflammatory clinical presentations of depression. Alongside improved novel biomarkers of LLD, it is suggested that earlier, improved diagnosis will contribute to tailored personalized care. The primary hope for medical/pharmacological treatment lies in the development of novel drugs, tailored and delivered according to the specific neuropathology identified within the phenotypic subtype of LLD. Undoubtedly, at the patient level, advances in the understanding of the biological processes driving the development and manifestation of LLD are critical to achieve this goal.

It is our opinion however, that a therapeutic pharmacological strategy, which fails to account for the wider perspective of successful aging, participation, purpose, meaning, function and sustained health, is most likely doomed to failure, when viewed from a larger perspective of prevention. Depression cannot, and must not, be viewed through the narrow lens of the purely medical paradigm. Rather it is incumbent upon not only healthcare professional in particular, but rather the cultural milieu in which they function, to reassess the perception of aging, and the role that people of advanced age partake within the fabric of society.

Addressing the numerous facets of loss which inevitably accompany advancing age must be prioritized to highlight the “social frailty” associated with aging in general and LLD in particular. Once recognized, action is required to address declining financial and social status; decreasing opportunities of employment, declining levels of volunteering or leisure activity; diminished social support with accompanying loneliness, isolation and lack of purpose. Ultimately, a recognition of the negative attitudes and stereotypes toward aging people, and toward aging itself is a necessary step toward positive change.

Health planners and policy makers, alongside concerned interest groups and stakeholders are indeed beginning to address such pressing issues as social isolation, age-associated poverty, lack of meaningful roles and activities available for older people. Novel interdisciplinary interventions spanning both medical, health, social and occupational related policies are needed to explore avenues of increased healthy and meaningful participation within society for older people. In addition to the reversal of stigma and negative perceptions, another approach is to acknowledge and honor the wisdom of older adults, accumulated throughout their lifetime. Such steps will only occur when ageism is itself confronted as an important obstacle in the way of health and successful aging.

The promotion of health behaviors aimed at increasing physical activity among older people is undoubtedly an important element in promoting improved mood, as well general mental and physical health. This would serve to stimulate a variety of elements critical in the process of successful aging, many elements of which fall within the domains of intrinsic capacity: locomotion, cognition, participation, vitality, as well as psychological and overall well-being. Indeed, physical activity, accompanied by coordinated dietary supplementation, is one of the very few proven interventions known to halt or reverse the phenotypic and biological markers of frailty (36).

Addressing the psychological needs of depressed individuals is often marginalized during the common medical interaction between the older depressed patient and their physician, and even when recognized, tends to be secondary to the initiation of anti-depressant medication. Yet, a considerable body of literature exists to support the existence and treatment of the biopsychosocial elements of LLD. Treatment should be directed at cognitive/psychological targets, promoting psychological resilience factors, as well as addressing social factors (37). Attempts to promote well-being, optimism, and hope are recently attracting more interest within the therapeutic context (38). Specifically, recent advances in the understanding of the psychology of hope, and the promotion of “hope intervention/therapy/training” have suggested numerous health benefits among older cancer patients. While it remains to be seen whether similar finding might be found for LLD, it nonetheless serves as an example for a growing interest in positive psychology and its influence upon health, longevity, aging and well-being. Interestingly, the modulating influence of hope upon muscle weakness measured using grip strength, a surrogate of frailty, with subsequent improved quality of life suggest that psychological and cognitive factors are intermediaries between physical frailty and long-term outcomes (39). Similarly, cognitive constructs such as “sense making theory” have been shown (40) to play a mediating role in the pathways between objective measures of illness burden, self-perception, and depression symptoms.

**Figure 1:**
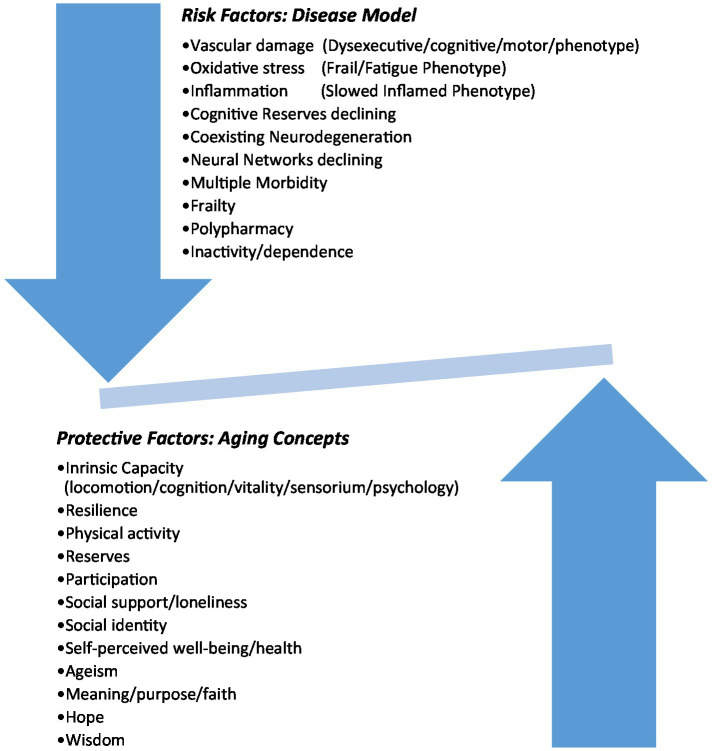
The balance of late life depression.

**Figure 2:**
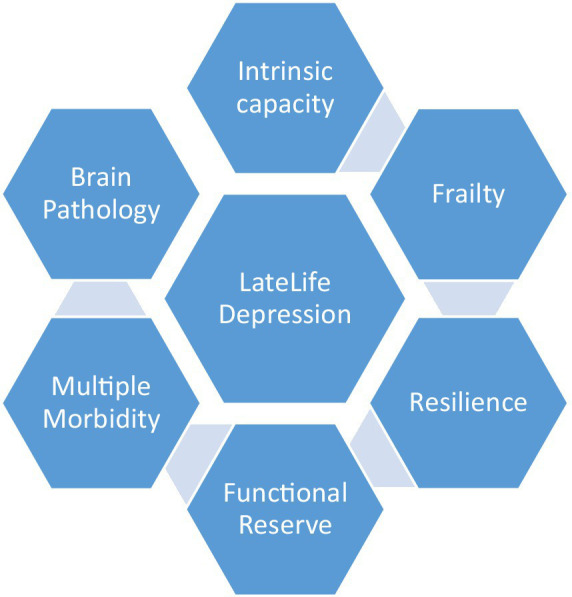
Late life depression against a background of aging domains.

In conclusion, late life depression presents a growing challenge to healthcare professionals, and exemplifies the need to expand contemporary conceptualization of age-related illnesses from a primarily medical model, toward a much wider and inclusive conceptualization of illness and health among older people themselves, and the aging process in general (Figures 1, 2). Our view of depression is framed within a generally more inclusive language of resilience, intrinsic capacity, and frailty; participation, inclusion and isolation; meaning, identity, and self-worth. Our paper clearly recognizes the necessity and importance of understanding the biology of depression at the individual treatment level. Nonetheless, in our opinion, a re-framing of depression within emerging theories and concepts of aging is suggested, as is a critical re-examination of the role of the older person in society.

## Data availability statement

The original contributions presented in the study are included in the article/supplementary material, further inquiries can be directed to the corresponding author.

## Author contributions

JJ, YR, GG, ES, and LB: conceptualization, original draft preparation, critical review and editing. All authors contributed to the article and approved the submitted version.

## Conflict of interest

The authors declare that the research was conducted in the absence of any commercial or financial relationships that could be construed as a potential conflict of interest.

## Publisher’s note

All claims expressed in this article are solely those of the authors and do not necessarily represent those of their affiliated organizations, or those of the publisher, the editors and the reviewers. Any product that may be evaluated in this article, or claim that may be made by its manufacturer, is not guaranteed or endorsed by the publisher.
